# Whole-Genome Resequencing Reveals Genetic Variation and Selection Signals in *Fusarium acuminatum* Causing *Astragalus* Root Rot

**DOI:** 10.3390/jof12070476

**Published:** 2026-06-30

**Authors:** Bingyan Xia, Jieyin Chen, Bin Ma, Xiaofeng Dai, Zhiqiang Kong

**Affiliations:** 1 State Key Laboratory for Biology of Plant Diseases and Insect Pests, Institute of Plant Protection, Chinese Academy of Agricultural Sciences, Beijing 100193, China; xiabingyan2001@163.com (B.X.); chenjieyin@caas.cn (J.C.); daixiaofeng_caas@126.com (X.D.); 2 Institute of Forestry and Grassland Ecology, Ningxia Academy of Agricultural and Forestry Sciences, Yinchuan 750002, China; mbin89@163.com

**Keywords:** *Fusarium acuminatum*, whole-genome resequencing, genetic variation, Ka/Ks, genetic differentiation

## Abstract

*Astragalus* root rot is a soil-borne disease primarily caused by *Fusarium* spp., which severely hampers the sustainable development of the *Astragalus* industry. *F. acuminatum* is a predominant pathogen causing this disease. To elucidate the genetic variation and adaptive evolutionary characteristics of *F. acuminatum* from different geographical origins, this study conducted whole-genome resequencing analysis on 28 isolates of *F. acuminatum* collected from four major *Astragalus* production regions. Approximately 124.9 Gb of high-quality sequencing data were obtained, and a large number of single-nucleotide polymorphisms (SNPs) were detected. Population genetic analysis revealed that strains from different regions did not form strictly geographically specific clusters, exhibiting a complex mixed distribution pattern. Nucleotide polymorphism analysis indicated that the Dingxi, Gansu (GD) population possessed the highest nucleotide diversity (π) value, reflecting the richest genetic diversity. Fixation index (Fst) analysis revealed significant genetic differentiation (Fst > 0.15) among populations from different provinces, suggesting that geographic isolation may be a contributing factor to restricted gene flow between pathogenic isolates in these regions. Tajima’s D positive values suggest a deviation from neutrality, consistent with balancing selection or population contraction. Ka/Ks analysis further revealed that the majority of genes exhibited Ka/Ks > 1, differing from the typical pattern of purifying selection dominance. This study revealed the genetic variation and selection signals of *F. acuminatum* isolates from different geographical origins, observed significant genetic differentiation between the Gansu and Ningxia populations, and identified a large number of genes that may be subject to positive selection.

## 1. Introduction

*Astragalus* is the dried root of *Astragalus membranaceus* (Fisch.) Bge. var. *mongholicus* (Bge.) Hsiao or *Astragalus membranaceus* (Fisch.) Bge. [1], a perennial herb and one of the most widely used medicinal herbs with high medicinal value, possessing various pharmacological activities such as immune enhancement, antioxidant activity, and anti-inflammatory effects [2,3]. However, in recent years, root rot caused by *Fusarium* spp. has posed a serious threat to high-quality *Astragalus* production. *Fusarium acuminatum* is one of the dominant pathogens and is distributed across major *Astragalus*-producing regions such as Gansu, Inner Mongolia, Shanxi, and Qinghai [4,5,6,7]. Elucidating the population genetic structure of a pathogen helps reveal its dispersal routes, gene flow patterns, and evolutionary dynamics, which are directly linked to disease emergence, spread, and persistence. Pathogen populations from different geographical origins may undergo genetic differentiation due to long-term adaptation to local environments, resulting in distinct ecotypes and virulence profiles [8]. This differentiation not only influences the geographical distribution of the disease but may also lead to the failure of control measures. Therefore, elucidating the population genetic structure and selection signals of the pathogen is of great significance for understanding the epidemiological patterns of the disease and formulating regional control strategies.

In recent years, population genomics has been widely applied to study plant pathogenic fungi, revealing their genetic structure, evolutionary history, and adaptive mechanisms at the whole-genome level [9]. Adaptive evolution in these fungi is often driven by genomic variations, including point mutations, insertion-deletion polymorphisms, and larger-scale structural variations [10,11]. Using these variations as molecular markers, whole-genome resequencing enables their genome-wide detection, providing unprecedented resolution for elucidating population structure and selection signals in pathogens. These methods have been successfully applied to studies of various plant pathogenic fungi. For example, Li et al. found through resequencing analysis of 38 isolates of *F. pseudograminearum* that high variability in secondary metabolite synthesis gene clusters was closely associated with the population’s geographic distribution and ecological adaptation [12]. Through resequencing of 60 isolates of *Exserohilum turcicum*, Cui et al. revealed host-driven population genetic differentiation and identified multiple candidate effector genes associated with host specificity [13]. Furthermore, Gladieux et al. conducted resequencing of 76 isolates of *Magnaporthe oryzae*, revealing the existence of multiple host-specific lineages and finding that gene flow between lineages significantly contributes to the genetic diversity of the pathogen [14]. These studies demonstrate that whole-genome resequencing has become a crucial approach for elucidating the population genetics and adaptive evolution of plant pathogenic fungi.

*F. acuminatum* is widely recognized as a major pathogen causing root rot on *Astragalus*. The genome of *F. acuminatum* was first sequenced in 2013 by Moolhuijzen et al. from wheat crown rot [15]. However, current population genetic studies on this fungus remain limited, and the genetic diversity, population structure, and adaptive evolutionary characteristics of isolates from different geographical origins remain unclear. To address this, this study collected *Astragalus* plants with root rot from four major *Astragalus*-producing regions, namely Dingxi and Gannan in Gansu, and Guyuan and Wuzhong in Ningxia, and performed whole-genome resequencing on 28 *F. acuminatum* strains isolated from these samples. This study aimed to: (1) systematically compare the genetic variation patterns, population structure, genetic diversity, and selection signals among different geographic populations of *F. acuminatum*; (2) identify candidate genes potentially under positive selection; and (3) provide preliminary insights into the genetic basis of regional adaptation in this pathogen.

## 2. Materials and Methods

### 2.1. Test Isolates and DNA Extraction

From July to September 2023, typical root rot samples were collected from fields with severe root rot outbreaks in the main *Astragalus* production areas of China. A total of 155 *Fusarium* isolates were obtained through the tissue isolation method [16]. Among them, 28 isolates were identified as *F. acuminatum* based on ITS and *TEF-1α* gene sequencing and used for subsequent whole-genome resequencing. The isolates were grouped into four populations based on their geographic origin: 10 isolates from Dingxi, Gansu (GD-1 to GD-10), 5 isolates from Gannan, Gansu (GG-1 to GG-5), 12 isolates from Guyuan, Ningxia (NG-1 to NG-12), and 1 isolate from Wuzhong, Ningxia (NW-1). Due to the limited sample size (n = 1), the NW isolate was not included in population-level analyses and was used solely for individual-level analyses.

The isolates were cultured on potato dextrose agar (PDA) at 25 °C for 7 days. Approximately 100 mg of mycelium was ground into a fine powder using liquid nitrogen, and genomic DNA was extracted using a modified CTAB method as described by Dar et al. [17]. The quality and concentration of the extracted DNA were assessed by agarose gel electrophoresis, Nanodrop spectrophotometry, and Qubit fluorometry.

### 2.2. Library Preparation and Genome Resequencing

After confirming DNA integrity by 1% agarose gel electrophoresis, the extracted DNA was randomly fragmented into approximately 350 bp fragments using a Covaris M220 focused ultrasonicator (Covaris, Inc., Woburn, MA, USA) with the following settings: peak power 50 W, duty factor 20%, cycles per burst 200, and treatment time 55 s. Libraries were constructed using the MGIEasy Universal DNA Library Preparation Kit (MGI Tech, Shenzhen, China) following the manufacturer’s protocol. The library preparation process included end repair, A-tailing, adapter ligation, purification, and PCR amplification. Library quality was assessed using an Agilent 2100 Bioanalyzer (Agilent Technologies, Santa Clara, CA, USA) to confirm the insert size (~350 bp) and the absence of adapter dimers. Library concentration was measured using a Qubit fluorometer (Thermo Fisher Scientific, Waltham, MA, USA) with the dsDNA HS Assay Kit. The library was diluted to 2 nM based on an average fragment size of 350 bp using the formula nM = (ng/µL × 10^6^)/(bp × 660), and sequenced on the DNBSEQ-T7 platform using paired-end 150 bp reads, targeting a sequencing depth of ≥50× per sample.

### 2.3. Data Processing and Sequence Alignment

Raw sequencing data were filtered using SOAPnuke (v1.5.6) [18] software to remove: (1) reads in which ≥50% of the bases had a quality score ≤ 20; (2) reads containing ≥ 1% N bases; (3) adapter contamination; and (4) PCR duplication contamination (i.e., duplicate reads generated during library amplification). This process generated clean data for subsequent analysis. The mem algorithm of BWA software (v0.7.17) [19] was used to align the clean reads to the *F. acuminatum* reference genome (GenBank accession: GCA_038181435.1). Sequencing depth and genome coverage were calculated using SAMtools (v1.12) depth and SAMtools stats commands.

### 2.4. Variant Detection and Annotation

Duplicate reads were first marked and removed using GATK (v4.0.9.0) MarkDuplicates. Base quality scores were then recalibrated using GATK BaseRecalibrator, followed by ApplyBQSR to apply the recalibration. SNPs and InDels were detected using GATK HaplotypeCaller [20]. Only reads with a mapping quality (MAPQ) ≥ 20 that were properly paired were retained for variant detection. following GATK best practice recommendations. For SNPs, variants were filtered out if they met any of the following criteria: QD < 2.0, FS > 60.0, MQ < 40.0, MQRankSum < −12.5, or ReadPosRankSum < −8.0. For InDels, variants were filtered out if they met: QD < 2.0, FS > 200.0, or ReadPosRankSum < −20.0. Only variants with FILTER = PASS were retained for downstream analyses.

The ANNOVAR software (version 20200607) [21] was used to perform functional annotation of the detected variant sites based on the *Fusarium acuminatum* reference genome annotation file (GFF, GenBank assembly accession: GCA_038181435.1). Annotation categories including the genomic region where the variant is located and the functional impact of variants in the coding region. Breakdancer (v1.4.5) [22] was used to detect structural variants (SVs), including chromosomal translocations (CTX), intrachromosomal translocations (ITX), inversions (INV), deletions (DEL), and insertions (INS).

### 2.5. Data Analysis

SNPs were filtered using PLINK (v1.90b6.21) [23], retaining loci with a minimum allele frequency (MAF) ≥ 0.05 and a genotype missing rate (geno) ≤ 0.2 for subsequent analysis. A maximum likelihood phylogenetic tree was constructed using SNPs and the IQ-TREE software (v2.3.2) based on the GTR model, with 1000 bootstrap repetitions [24]. Principal component analysis (PCA) was performed using the GCTA software (v1.94.1). A phylogenetic matrix was constructed based on the SNP data, and the first three principal components were calculated [25]. Population structure analysis was conducted using the Admixture software (v1.3.0) [26], with K values ranging from 1 to 10; the optimal K value was determined using cross-validation (CV) error. The average Linkage disequilibrium (LD) coefficient r^2^ was calculated using the PopLDdecay software (v3.41) [27], and LD decay plots were generated using the Plot_MutiPop.pl script within the PopLDdecay package. Vcftools (v0.1.16) [28] was used to calculate nucleotide diversity (π) and Tajima’s D values using 20 kb windows and 10 kb steps. Fst values between pairs of populations were calculated using the method of Weir and Cockerham (1984) [29]. Codon alignments of SNP-containing CDS sequences were derived from the reference genome annotation file, and the KaKs Calculator (v2.0) [30] was used to calculate the Ka/Ks values for each gene across the entire population based on the MS model.

## 3. Results

### 3.1. Data Quality Assessment and Comparison Results

Whole-genome resequencing was performed on 28 strains of *F. acuminatum*, yielding a total of 832.33 million high-quality clean reads, amounting to 124.85 Gb of clean bases, with an average of approximately 4.46 Gb of data generated per sample. Quality assessment results showed that the Q20 score for all samples was no less than 96.74%, the Q30 score was no less than 91.19%, and the average GC content was 47.93%, indicating that the sequencing data quality was reliable. Alignment of the clean reads to the *F. acuminatum* reference genome revealed that the mapping coverage for each sample ranged from 89.25% to 97.52%, with an average coverage of 95.20%, indicating a high degree of homology between the reference genome and the sequenced samples. Genome coverage analysis showed that the average sequencing depth of the samples was 80.45×, with an average coverage of 1× at 92.8% and an average coverage of 4× at 92.0%. These values were comparable, indicating that only approximately 0.8% of the genome is in a state of low-depth coverage. These results fully demonstrate that the sequencing data quality is good, with both coverage and depth meeting the requirements for subsequent SNP/InDel detection and population genetic analysis, and detailed statistics in Supplementary Table S1.

### 3.2. Genome-Wide Variation Analysis

#### 3.2.1. Overall Statistics for SNPs, Indels, and SVs

**Figure 1:**
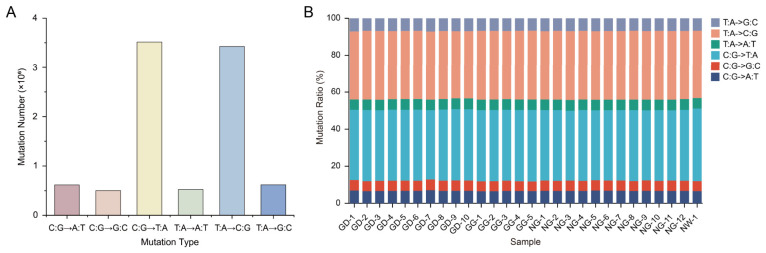
Mutation spectrum of SNP types. (**A**) Mutation spectrum of SNP types across all samples; (**B**) Mutation spectrum of SNP types for each individual sample. The six substitution types are labeled as C:G→A:T, C:G→G:C, C:G→T:A, T:A→A:T, T:A→C:G, and T:A→G:C (arrow: reference → variant).

Variation analysis was conducted on 28 isolates of *F. acuminatum* based on the ~46.7 Mb reference genome. After rigorous quality control filtering, the statistical results for variation sites in each sample are shown in Supplementary Table S2. A total of 9,205,729 SNP sites were detected across all samples, with an average of 328,776 SNPs per sample. When classifying all SNPs into six base substitution types, a total of 6,938,927 transition events and 2,256,752 transversion events were detected, with a transition-to-transversion ratio (Ti/Tv) of 3.07. This indicates that transitions dominate, while transversions account for a lower proportion (Figure 1A). Specifically, the C:G→T:A transition was the most abundant (3,514,537, accounting for 38.2% of all SNPs), followed by the T:A→C:G transition (3,423,890, accounting for 37.2%), together, these two accounted for 75.4% of all SNPs. Among transversions, the C:G→A:T, T:A→G:C, C:G→G:C, and T:A→A:T were relatively balanced. The mutation spectrum was consistent across all individual samples (Figure 1B). Across the entire genome, a total of 524,244 InDel sites were detected, with an average of 18,723 InDels per sample. Among the InDels, the ratio of insertions (Insert) to deletions (Delete) was essentially balanced, at approximately 0.97.

In addition, a total of 388 SV events were detected across the entire genome, including three types: interchromosomal translocations (CTX), intrachromosomal translocations (ITX), and inversions (INV). Among these, ITX was the most abundant (274, accounting for 74.3%), followed by INV (94, accounting for 25.5%), while only one CTX was detected (0.2%). The number of SVs varied significantly among samples, ranging from 0 to 62. Sample GG-3 exhibited the highest number of SVs (62), whereas four samples (GD4, GD6, GD7, and NG2) had no SVs detected.

#### 3.2.2. Genomic Distribution and Functional Annotation of SNPs and Indels

**Figure 2:**
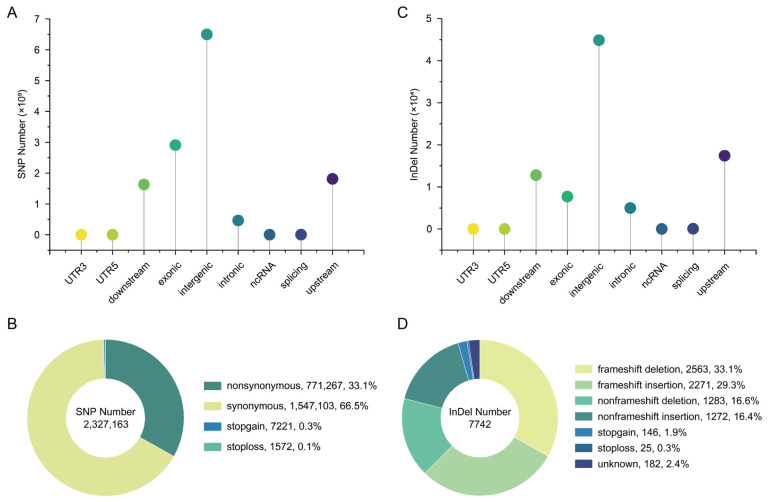
Genomic distribution and functional annotation of SNPs and InDels. (**A**) Distribution proportion of SNPs across different genomic regions. UTR3/UTR5: variant located in the 3′ or 5′ untranslated region; Upstream/Downstream: Variants within 1 kb upstream of a gene’s transcription start site (TSS) or downstream of its transcription termination site (TTS); Exonic: variant located in a coding exon; Intergenic: variant located between genes; Intronic: variant located in an intron; ncRNA: variant located in a non-coding RNA gene; Splicing: variant within 2 bp upstream of a splice site (non-exonic region); (**B**) Functional annotation of SNPs in coding regions; (**C**) Distribution proportion of InDels across different genomic regions (definitions as in A); (**D**) Functional annotation of InDels in coding regions.

Genomic region annotation of SNP loci across the entire genome revealed that SNPs are unevenly distributed across different regions. Intergenic regions accounted for the highest proportion, reaching 46.67%; followed by exonic regions (22.74%), upstream regions (14.16%), and downstream regions (12.75%); intronic regions accounted for only 3.63%, while other regions (UTR, splice sites, ncRNA, etc.) collectively accounted for less than 0.1% (Figure 2A). Functional annotation of SNPs in exonic regions was performed, categorizing them into four types based on their impact on protein coding: synonymous SNPs, nonsynonymous SNPs, stop-gain, and stop-loss. The results showed that synonymous mutations were the most abundant, accounting for 66.5% of SNPs in exonic regions; nonsynonymous mutations were the next most common, accounting for 33.1% (Figure 2B).

The distribution pattern of Indels is similar to that of SNPs, with the majority located in intergenic regions (44.32% of all Indels), followed by upstream regions (19.80%) and downstream regions (14.55%) (Figure 2C). Functional annotation of InDels in exonic regions was performed, and they were categorized based on their impact on protein coding into frameshift deletions, frameshift insertions, non-frameshift deletions, non-frameshift insertions, stop-gain, and stop-loss, among other types. The results showed that frameshift mutations were the predominant type of InDels in the coding region, totaling 4834 and accounting for 62.43% of all exonic InDels; non-frameshift mutations (non-frameshift deletions + non-frameshift insertions) totaled 2555, accounting for 33.00% (Figure 2D).

### 3.3. Analysis of Population Genetic Structure

#### 3.3.1. Phylogenetic Analysis

**Figure 3:**
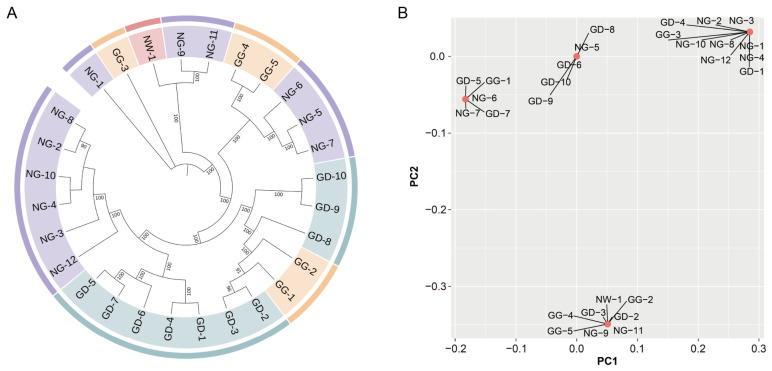
Phylogenetic tree and principal component analysis of *F. acuminatum*. (**A**) Maximum likelihood phylogenetic tree constructed based on genome-wide SNPs, with bootstrap values (≥70%) indicated at branch nodes; (**B**) Principal component analysis (PCA) plot of 28 isolates, with PC1 and PC2 representing the first and second principal components, respectively.

The results of phylogenetic relationships showed that the 28 isolates formed multiple branches on the phylogenetic tree (Figure 3A). At the population level, the GD population clustered into two adjacent branches, indicating close phylogenetic relationships and a relatively homogeneous structure within this group. The NG population was more dispersed, clustering into three separate branches, with one strain forming an independent branch, indicating high internal genetic diversity. The distribution of the GG population was the most complex: two isolates clustered within the NG group, two within the GD population, and one formed an independent branch. The NW-1 isolate clustered with the NG group on the same branch. It is worth noting that although distinct subclusters exist within the isolates of each group, they do not form independent branches on the phylogenetic tree strictly according to geographic origin. Instead, they exhibit clear cross-geographic clustering. The results of the principal component analysis were consistent with those of the phylogenetic analysis, with the 28 isolates broadly grouped into four clusters (Figure 3B). This mixed clustering pattern indicates genetic similarity and a lack of strict geographic structuring among *F. acuminatum* populations from different geographic origins.

#### 3.3.2. Analysis of Genetic Structure

**Figure 4:**
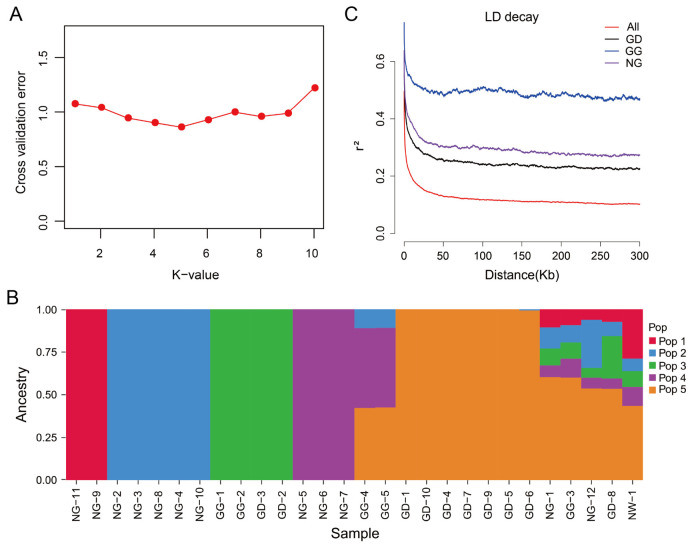
Population structure and linkage disequilibrium analysis of *F. acuminatum*. (**A**) Cross-validation (CV) error curve for different K values, with the minimum CV error observed at K = 5; (**B**) Population structure stacked bar plot of 28 isolates at K = 5, where each column represents an isolate, different colors represent distinct ancestral components, and the length of each colored segment indicates the proportion of the corresponding ancestral component; (**C**) Linkage disequilibrium (LD) decay curves of different populations.

Cluster analysis revealed that the CV error was minimal when K = 5, suggesting that the 28 isolates of *F. acuminatum* could be preliminarily classified into 5 optimal ancestral components (Figure 4A). When K = 5, the ancestral composition varied among samples (Figure 4B). Specifically, five isolates (NG-1, NG-12, GD-8, GG-1, and NW-1) showed contributions from all five ancestral components (mixed ancestry); two isolates (GG-4 and GG-5) showed contributions from three ancestral components; and the remaining 21 isolates were predominantly derived from a single ancestral component. It is worth noting that the ancestral component types differ among strains from the same geographic source, indicating that the populations are not genetically homogeneous but exhibit a certain degree of inter-individual variation. These results indicate the presence of genetic admixture both within and among *F. acuminatum* populations, and suggest that isolates from the same geographic origin can have distinct genetic backgrounds.

LD decay analysis was performed on the GD, GG, and NG populations. The results showed significant differences in the LD decay patterns among the populations (Figure 4C). The GD population exhibited the longest LD decay distance, with r^2^ decaying to approximately 0.25 before stabilizing; the NG population showed an intermediate decay pattern, stabilizing at r^2^ of approximately 0.30; and the GG population had the shortest decay distance, stabilizing at the highest r^2^ of approximately 0.50. These differences reflect variations in the evolutionary histories of different geographic populations.

### 3.4. Genetic Diversity and Differentiation

**Figure 5:**
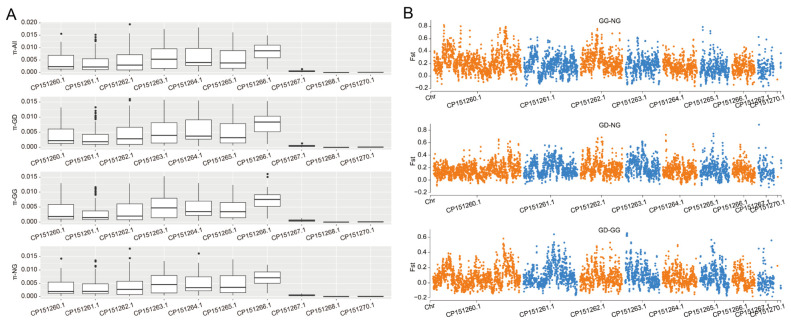
Chromosomal distribution of π values and pairwise Fst among populations. (**A**) Boxplots of π value distribution across chromosomes for each population, where the box represents the interquartile range and the horizontal line inside the box indicates the median; (**B**) Genome-wide distribution of pairwise Fst values, with comparisons shown from top to bottom as GG vs. NG, GD vs. NG, and GD vs. GG.

To assess the levels of genetic diversity among different geographic populations of *F. acuminatum*, the whole-genome nucleotide diversity (π) was calculated for each population. The results showed that the GD population had the highest average π value (0.004359), indicating the greatest genetic diversity; the NG population ranked second (0.004108) and also exhibited high genetic diversity; the average π value of the GG population was slightly lower than that of the GD and NG populations (0.003955). Further analysis of the distribution of π values across different scaffolds revealed that the distribution trends of the π values in the GD, GG, and NG populations were generally consistent, but there were significant differences among the individual scaffolds (Figure 5A). Among them, the median π value of scaffolds CP151266.1 was significantly higher than that of the others, indicating that this chromosome possesses the richest genetic diversity; the presence of numerous outliers on scaffolds CP151261.1 and CP151262.1 suggests that π values in certain regions of these scaffolds deviate significantly from the genomic background, potentially indicating signals of selective sweep.

Pairwise Fst values were calculated for GD vs. GG, GD vs. NG, and GG vs. NG (Figure 5B). The average Fst for GD vs. GG was 0.076, indicating moderate genetic differentiation (commonly defined as Fst between 0.05 and 0.15); the average Fst for GD vs. NG was 0.173, indicating high genetic differentiation (Fst > 0.15); and the average Fst for GG vs. NG was 0.189, also indicating high genetic differentiation. The genetic differentiation between the two Gansu subpopulations (GD vs. GG) was the lowest, suggesting that the *F. acuminatum* populations in Dingxi and Gannan are closely related and may exhibit potential gene flow. In contrast, the populations of Gansu and Ningxia (GD vs. NG, GG vs. NG) both exhibited high genetic differentiation (Fst > 0.15), suggesting the presence of a significant genetic barrier between Gansu and Ningxia. Geographical isolation may be a contributing factor to restricted gene flow between the pathogenic strains in these two regions.

### 3.5. Selection Pressure Analysis

**Figure 6:**
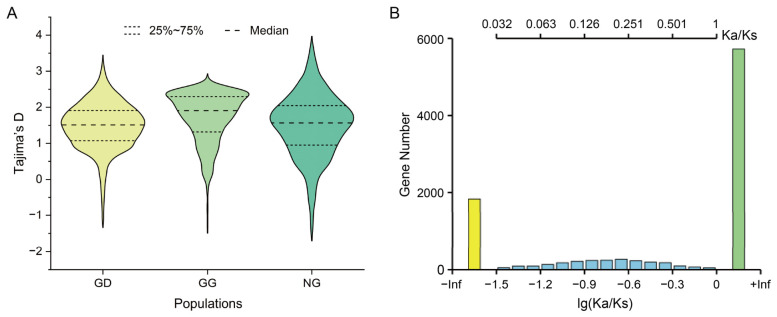
Tajima’s D distribution across populations and genome-wide Ka/Ks frequency distribution. (**A**) Violin plots of Tajima’s D distribution for each population, where the width of the violin represents the probability density; (**B**) Genome-wide Ka/Ks frequency distribution, with log10(Ka/Ks) < 0 indicating purifying selection and log10(Ka/Ks) > 0 indicating positive selection.

Tajima’s D values were calculated for each population. The results showed that the mean Tajima’s D values for all populations were positive, with the GG population having the highest mean (1.730), followed by the NG population (1.488), and the GD population having the lowest (1.477) (Figure 6A). The Tajima’s D values for all populations were greater than 0, suggesting that balancing selection or population contraction may be occurring.

To assess the selective pressures acting on the protein-coding genes of *F. acuminatum*, the CDS sequences containing SNP mutations were extracted from the gene regions of each sample, Ka and Ks values were calculated for each gene, and the Ka/Ks ratios were obtained. The results showed that among the detected genes, 4096 had a Ka/Ks ratio < 1, while 5798 had a Ka/Ks ratio > 1 (Figure 6B). Notably, the number of genes with Ka/Ks > 1 exceeded that with Ka/Ks < 1, a pattern that differs from the typical characteristic of purifying selection dominance observed in most genomes, suggesting that they may be under positive selection pressure.

## 4. Discussion

*F. acuminatum* is a soil-borne pathogen with a broad host range; in addition to causing damage to *Astragalus*, it can also infect various medicinal plants such as *Panax ginseng*, *Angelica sinensis*, *Schisandra chinensis*, and *Medicago sativa* [31,32,33,34], causing root rot, plant wilting, and even death. In severe cases, it can lead to widespread yield losses, resulting in significant economic losses for the medicinal plant industry [35]. Furthermore, *F. acuminatum* can also infect important field crops such as soybeans, wheat, and potatoes [36,37,38]. Its broad host range indicates that *F. acuminatum* holds significant agricultural and economic importance. In-depth research into its population genetic structure and adaptive evolutionary mechanisms provides important theoretical guidance for the regional prevention and control of various crop diseases. This study represents the first application of whole-genome resequencing technology to the population genetics of *F. acuminatum*, the pathogen responsible for *Astragalus* root rot. By detecting SNP, InDel, and SV variations across the entire genome and combining these with multidimensional methods such as phylogenetic analysis, genetic variation analysis, and selection pressure analysis, the population genetic characteristics of this pathogen have been preliminarily elucidated.

The consistency between multiple analytical approaches strengthens our conclusions. First, the LD decay patterns align with the π results: the GD population, which showed the longest LD decay distance (indicating stronger linkage), also exhibited the highest genetic diversity (π = 0.00436), while the GG population, with the shortest LD decay distance (indicating higher recombination rate), showed the lowest π value (0.00395). While selective sweeps in GD and higher recombination in GG could explain these patterns, alternative interpretations such as differences in effective population size or demographic history, cannot be excluded. Second, the positive Tajima’s D values across all populations (1.48–1.73) are consistent with either balancing selection or population contraction. This finding parallels observations from the rice false smut pathogen Ustilaginoidea virens [39], where similarly positive Tajima’s D values and Fst values exceeding 0.15 were observed, suggesting that both pathogens may have experienced comparable evolutionary pressures. Third, Ka/Ks analysis revealed an unusual result: Ka/Ks > 1 genes were predominant (5798 vs. 4096 for Ka/Ks < 1). In most biological genomes, purifying selection dominates to maintain essential functions. The high proportion of Ka/Ks > 1 genes in *F. acuminatum* raises the possibility of rapid adaptive evolution, potentially related to pathogenicity and host adaptation. However, several factors should be considered when interpreting this pattern. Slightly deleterious mutations segregating at low frequencies may inflate Ka/Ks estimates, and demographic processes (e.g., population contraction) can also affect the ratio. Therefore, functional validation of candidate genes remains necessary to confirm genuine positive selection.

Nevertheless, several limitations should be acknowledged. First, the sample sizes were unbalanced among populations, and the small sample sizes of the NW (n = 1) isolate and GG (n = 5) populations limit the statistical power of population-level estimates. The GG population results should be interpreted with caution. Second, the reference genome used is currently at the scaffold level; a chromosome-level assembly would improve the precision of genomic distribution analyses for π and Tajima’s D. Third, functional validation of candidate positively selected genes is needed to confirm their adaptive roles. Future studies should expand sampling from the GG and NW regions, obtain a chromosome-level genome assembly, and perform experimental validation of selected candidate genes to further elucidate the adaptive mechanisms of this pathogen. In summary, our multi-faceted analysis—integrating phylogenetics, population structure, LD decay, nucleotide diversity, Tajima’s D, Fst, and Ka/Ks—reveals that *F. acuminatum* isolates from Gansu and Ningxia exhibit significant genetic differentiation, likely due to geographical isolation, and show evidence of rapid adaptive evolution on genes associated with pathogenicity and host adaptation. These findings provide a theoretical foundation for region-specific disease management and resistance breeding.

## 5. Conclusions

This study systematically revealed the population genetic structure and signals of natural selection in *F. acuminatum* populations from Gansu and Ningxia using whole-genome resequencing technology. Whole-genome variant analysis identified a total of 9,205,729 SNPs, 524,244 InDels, and 388 SVs. Population genetic analysis revealed that isolates from different geographic origins did not form strictly geographically specific clusters in the phylogenetic tree or PCA, exhibiting a complex pattern of mixed distribution. Population structure analysis indicated an optimal K value of 5, with varying degrees of admixture among populations. LD decay analysis showed significant differences in decay rates among populations, with the GD population exhibiting a longer LD decay distance, while the GG population showed the shortest decay distance. Genetic diversity analysis indicated that the GD population had the highest nucleotide diversity (π = 0.00436), followed by the NG population (π = 0.00411). Fst analysis revealed moderate differentiation between GD and GG (Fst = 0.076), but high differentiation between NG and GG (Fst = 0.189) as well as between GD and NG (Fst = 0.173), suggesting a significant genetic barrier may exist between the Gansu and Ningxia populations. The average Tajima’s D value was positive across all populations (1.48–1.73), consistent with either balancing selection or population contraction. Ka/Ks analysis revealed that genes with Ka/Ks > 1 (5798) were more numerous than those with Ka/Ks < 1 (4096). This study elucidates the genetic variation and adaptive evolutionary characteristics of *F. acuminatum*, providing a theoretical basis for understanding its mechanisms of regional adaptation and population dynamics. The findings advance our understanding of how geographic isolation and selection shape pathogen diversity in *F. acuminatum*.

## Data Availability

The raw sequencing data have been deposited in the NCBI Sequence Read Archive (SRA) under BioProject accession number PRJNA1480852. All other data are included in the manuscript.

## Supplementary Materials

The following supporting information can be downloaded at: https://www.mdpi.com/article/10.3390/jof12070476/s1, Table S1: Sequencing Data Quality and Alignment Statistics for all 28 *F. acuminatum* isolates; Table S2: Statistics on SNPs, InDels, and SVs for Each Sample.
