# Supplementary material for: Whole-Genome Resequencing Reveals Genetic Variation and Selection Signals in Fusarium acuminatum Causing Astragalus Root Rot
Source: J Fungi (Basel). 2026 Jun 30;12(7):476. doi: 10.3390/jof12070476 (PMC13412694; doi:10.3390/jof12070476)
Supplement: Supplementary file 1 [file jof-12-00476-s001.zip › jof-4321122-supplementary.pdf]

Supplementary Table S1. Sequencing Data Quality and Alignment Statistics for all 28 *F. acuminatum* isolates.

Sample	Clean reads (M)	Total Base (Gb)	GC (%)	Q30 (%)	Mapping rate (%)	Average depth (X)	Coverage 4X (%)
GD-1	42.93	6.44	49.42	95.67	97.52	123.79	87.45%
GD-2	27.59	4.14	48.03	95.44	94.23	78.80	93.23%
GD-3	16.70	2.51	48.06	95.19	94.58	47.92	92.61%
GD-4	26.66	4.00	47.80	94.08	95.68	63.03	88.76%
GD-5	10.02	1.50	47.54	92.94	93.34	28.53	92.36%
GD-6	26.92	4.04	48.17	95.40	94.96	77.98	89.94%
GD-7	15.67	2.35	48.83	95.52	95.00	44.98	91.06%
GD-8	20.28	3.04	48.26	95.43	96.65	59.60	88.46%
GD-9	14.26	2.14	47.44	93.05	91.64	40.01	88.92%
GD-10	23.84	3.58	47.94	95.07	95.44	69.97	89.19%
GG-1	37.72	5.66	47.93	92.34	95.16	99.44	93.43%
GG-2	33.95	5.09	47.93	91.35	94.93	87.07	93.42%
GG-3	39.50	5.93	48.01	94.92	93.72	98.90	93.11%
GG-4	31.06	4.66	48.15	91.53	96.75	83.98	88.83%
GG-5	42.93	6.44	48.05	93.62	97.28	111.68	88.99%
NG-1	29.34	4.40	48.27	91.77	96.55	96.07	93.53%
NG-2	21.72	3.26	48.07	92.07	96.94	64.67	93.45%
NG-3	24.57	3.69	48.23	91.47	89.32	60.30	93.96%
NG-4	23.76	3.56	47.95	91.19	97.48	62.80	93.74%
NG-5	20.33	3.05	47.27	94.84	89.25	55.26	91.99%
NG-6	38.67	5.80	47.96	93.97	96.76	102.47	92.66%
NG-7	33.22	4.98	48.03	94.17	97.19	89.60	92.52%
NG-8	42.93	6.44	48.27	92.75	96.68	115.01	93.97%
NG-9	40.62	6.09	47.88	94.24	95.08	104.24	94.43%
NG-10	39.41	5.91	48.19	92.01	95.95	77.55	93.93%
NG-11	37.92	5.69	47.85	94.01	97.45	105.67	93.61%
NG-12	37.17	5.58	48.19	94.92	94.35	108.49	94.43%
NW-1	32.61	4.89	48.22	95.56	95.84	94.76	94.26%

Supplementary Table S2. Statistics on SNPs, InDels, and SVs for Each Sample.

Sample	SNP			InDel			SV			
	Total	Transition	Transversion	Total	Insert	Delete	Total	CTX	INV	ITX
GD-1	297040	222526	74514	16868	8343	8525	1	0	0	1
GD-2	339156	256967	82189	19336	9463	9873	2	0	0	2
GD-3	330852	249948	80904	19001	9316	9685	1	0	0	1
GD-4	313503	236484	77019	17492	8605	8887	0	0	0	0
GD-5	356002	267919	88083	20315	9966	10349	2	0	1	1
GD-6	317492	239631	77861	18070	8824	9246	0	0	0	0
GD-7	331766	247126	84640	19438	9600	9838	0	0	0	0
GD-8	331125	249680	81445	19289	9555	9734	1	0	0	1
GD-9	335660	251653	84007	19488	9526	9962	2	0	1	1
GD-10	337733	253355	84378	19614	9593	10021	9	0	6	3
GG-1	333852	252963	80889	18923	9241	9682	33	0	5	28
GG-2	332657	251779	80878	18900	9240	9660	32	0	6	26
GG-3	365874	275556	90318	20554	10146	10408	62	0	4	58
GG-4	320066	242597	77469	18055	8903	9152	25	0	5	20
GG-5	322248	244662	77586	18145	8948	9197	11	0	3	8
NG-1	342681	257326	85355	19499	9642	9857	31	0	6	25
NG-2	330697	249980	80717	18727	9231	9496	0	0	0	0
NG-3	346103	260160	85943	19467	9660	9807	26	0	14	12
NG-4	328310	247979	80331	18516	9094	9422	6	0	2	4
NG-5	298717	224172	74545	17159	8374	8785	2	0	0	2
NG-6	304333	229114	75219	17256	8438	8818	15	0	1	14
NG-7	301026	226478	74548	17090	8369	8721	17	0	4	13
NG-8	331924	251272	80652	18696	9239	9457	18	0	7	11
NG-9	334552	251191	83361	19040	9294	9746	35	1	10	24
NG-10	328082	248098	79984	18491	9142	9349	22	0	4	18
NG-11	344797	259362	85435	19959	9780	10179	24	0	15	9
NG-12	303153	228402	74751	17427	8576	8851	8	0	1	7
NW-1	346328	262547	83781	19429	9532	9897	3	0	1	2
